# Predictors of complication after adrenalectomy

**DOI:** 10.1590/S1677-5538.IBJU.2018.0482

**Published:** 2019-07-27

**Authors:** Victor Srougi, João A. B. Barbosa, Isaac Massaud, Isadora P. Cavalcante, Fabio Y. Tanno, Madson Q. Almeida, Miguel Srougi, Maria C. Fragoso, José L. Chambô

**Affiliations:** 1 Divisão de Urologia da Faculdade de Medicina da Universidade de São Paulo, São Paulo, Brasil; 2 Divisão de Endocrinologia da Faculdade de Medicina da Universidade de São Paulo, São Paulo, Brasil

**Keywords:** Adrenalectomy, Morbidity, Pathology

## Abstract

**Purpose:**

To investigate risk factors for complications in patients undergoing adrenalectomy.

**Materials and Methods:**

A retrospective search of our institutional database was performed of patients who underwent adrenalectomy, between 2014 and 2018. Clinical parameters and adrenal disorder characteristics were assessed and correlated to intra and post-operative course. Complications were analyzed within 30-days after surgery. A logistic regression was performed in order to identify independent predictors of morbidity in patients after adrenalectomy.

**Results:**

The files of 154 patients were reviewed. Median age and Body Mass Index (BMI) were 52-years and 27.8kg/m^2^, respectively. Mean tumor size was 4.9±4cm. Median surgery duration and estimated blood loss were 140min and 50mL, respectively. There were six conversions to open surgery. Minor and major post-operative complications occurred in 17.5% and 8.4% of the patients. Intra-operative complications occurred in 26.6% of the patients. Four patients died. Mean hospitalization duration was 4-days (Interquartile Range: 3-8). Patients age (p=0.004), comorbidities (p=0.003) and pathological diagnosis (p=0.003) were independent predictors of post-operative complications. Tumor size (p<0.001) and BMI (p=0.009) were independent predictors of intra-operative complications. Pathological diagnosis (p<0.001) and Charlson score (p=0.013) were independent predictors of death.

**Conclusion:**

Diligent care is needed with older patients, with multiple comorbidities and harboring unfavorable adrenal disorders (adrenocortical carcinoma and pheocromocytoma), who have greater risk of post-operative complications. Patients with elevated BMI and larger tumors have higher risk of intra, but not of post-operative complications.

## INTRODUCTION

Adrenal disorders are found in 5% of the population and this rate increases with age (1). Although the majority of the patients will not need surgical intervention, adrenal glands can harbor a variety of pathologies that may originate life threating clinical conditions. Probably due to advances in diagnostic exams, the number of adrenalectomies performed in the last decades increased approximately 65% (2). Furthermore, the demystification of adrenal surgery may have contributed to augment this number. Of noteworthy, four decades ago, morbidity and mortality after adrenalectomy were as high as 30% and 4%, respectively (3). Lately, contemporary series reports complication rates varying from 6-18% and mortality <1% (2, 4, 5). Despite an important reduction in morbidity, the rate of complications is still high. Adrenal disorders are heterogeneous and thus convalescence should be expected to be uneven. Identifying clusters of patients at higher risk of complications would be valuable for peri-operative preparation and enhancement of surgical strategy. Our objective is to find predictors of surgical and clinical complications after adrenalectomy for the treatment of different etiologies.

## MATERIALS AND METHODS

A retrospective review of our prospective collected database was performed searching for patients who underwent total or partial adrenalectomy, between July of 2014 and January of 2018. Inclusion criteria comprised adult patients, bearing any primary adrenal tumor etiology, paraganglioma or secondary Cushing's syndrome (pituitary or ectopic neuroendocrine tumor), who underwent open or trans-peritoneal laparoscopic access. Patients indicated to adrenalectomy that were intra or post-operatively diagnosed with tumors of other organs or patients with incomplete data were excluded. Data collection included a thorough recording of comorbidities and classification according to the Charslon index (6). All procedures were performed by the same experienced surgeon, who performs >40 cases yearly for at least 10 years, teaching urology residents.

Indication to adrenalectomy were (1) hormone producing tumors and (2) tumors >4cm. Hormonal investigation was done by discrimination of the endocrinologist and all patients underwent abdominal computed tomography or magnetic resonance imaging. Patients were prepared to surgery following our institution guidelines and according to their adrenal disorder (Table-1). Open access was chosen for all tumors >8cm and most tumors >6cm. Patients with unilateral adrenal tumors were indicated to total ipsilateral adrenalectomy. Patients harboring synchronic bilateral adrenal tumors or with refractory Cushing's disease underwent bilateral adrenalectomy; total or partial resection of the gland was chosen depending on the pathology. Laparoscopic total or partial adrenalectomy were performed as described elsewhere (7).

**Table 1:** Pre-operative preparation.

Adrenal disease	Preparation
Hiperaldosteronism	Oral Spironolactone when K <3.5 and BP >140×90 mmHg
Pheocromocytoma	Oral Prazosin until mean BP is between 65-90 mmHg Oral beta-blocker if heart rate >100 1000mL of saline 2h before surgery
ACC	Sub-cutaneous Enoxaparin until 12h before surgery Meningococcal, Pneumococcal and *Haemophilus influenzae* vaccination if splenectomy needed
Cortisol producing adenoma, Cushing's disease and PMAH	Sub-cutaneous Enoxaparin until 12h before surgery, 100 mg of intra-venous Hydrocortisone in anesthetic induction.

**BP** = blood pressure; **PMAH** = primary macronodular adrenal hyperplasia; **ACC** = adrenocortical carcinoma.

Demographic data, comorbidities, clinical presentation, tumor etiology and size were assessed. Outcome measures were surgery duration, intra-operative complications, intra-operative blood loss, intra-operative hemodynamic instability, conversion to open surgery (in case of laparoscopic adrenalectomy) and 30-day post-operative complications. Complications were categorized according to the Clavien-Dindo classification (8). Intra-operative complications were classified according to the system proposed by Satava (9). Hemodynamic instability was considered when any vasoactive drug was needed to regulate blood pressure.

Statistical analysis was performed using SPSS Statistics 22.0 (IBM, Armonk NY). Univariate analysis was performed to evaluate the association between independent variables with surgical and postoperative outcomes. Pathological diagnosis was categorized as adrenocortical carcinoma (ACC), pheocromocytoma and others, in order to perform the assessment. Categorical variables were analyzed using chi-square and ANOVA tests. Continuous variables were analyzed using Student's t-test and Mann-Whitney U test. Kruskal-Wallis test was performed in cases of multiples categories. Analysis of association between two continuous variables was performed with Pearson's bivariate correlation. Variables significantly associated with each outcome on univariate analysis were included in a model of multivariate analysis. For categorical outcomes, a Binomial Logistic Regression was performed; for continuous variables, a Multivariate Linear Regression was performed. Results were considered statistically significant at p<0.05; a two-tailed test was used whenever applicable.

## RESULTS

In the study period 162 patients were indicated to adrenalectomy at our institution, of whom 154 patients met the inclusion criteria and were analyzed (8 patients excluded due to final pathological diagnosis of retroperitoneal sarcoma, renal cell carcinoma and hepatic carcinoma). Incidentalomas represented 20% of diagnoses. Twenty-five patients with large adrenal masses had clinical suspicion of ACC; eleven had pathological confirmation (Table-2). Hyperaldosteronism (25%) and pheocromocytoma (21%) were the most prevalent adrenal disorders. The frequencies of other pathologies are discriminated in Tables 3 and 4.

**Table 2:** Final pathology of large adrenal masses suspect of adrenocortical carcinoma at diagnosis (N=25).

Final Pathology	N (%)
ACC	11 (44%)
Adenoma	7 (28%)
Mielolipoma	2 (8%)
Hemangioma	2 (8%)
Ganglioneuroma	1 (4%)
Schwanoma	1 (4%)
*Paracoccidioides brasiliensis* infecction	1 (4%)

**ACC** = adrenocortical carcinoma

**Table 3:** Demographic and clinical presentation characteristics.

Parameters		Mean ± SD	Median (IQR)
	Age (years)	50.8±14.9	52 (40-62)
	Sex, males	52 (33.8%)	
**Side**			
	Right (%)	64 (41.5%)	
	Left (%)	62 (40.3%)	
	Bilateral (%)	28 (18.2%)	
BMI (kg/m^2^)		29.2±6.7	27.8 (25-33)
**Charlson Score**			
	0	48 (31.2%)	
	1-5	89 (57.8%)	
	5-10	15 (9.7%)	
	>10	2 (1.3%)	
**Clinical presentation**			
	Incidental	31 (20.1%)	
	Blood hypertension	68 (44.2%)	
	Cushing syndrome	29 (18.8%)	
	Hypertensive crisis	17 (11%)	
	Pain	13 (8.4%)	
	Palpable mass	8 (5.2%	
	Virilization	5 (3.2%)	
**Pre-operative diagnosis**			
	Aldosterone producing adenoma	39 (25.3%)	
	Pheocromocytoma	33 (21.4%)	
	PMAH	16 (10.5%)	
	Cortisol producing adenoma	15 (9.7%)	
	Cushing's disease	15 (9.7%)	
	ACC	25 (16.2%)	
	Paraganglioma	4 (2.6%)	
	Myelolipoma	2 (1.3%)	
	Metastasis	2 (1.3%)	
	Cyst	2 (1.3%)	
	Ganglioneuroma	1 (0.7%)	
Tumor size, (cm)		4.9±4	3.4 (2-6.5)

**BMI** = body mass index; **PMAH** = primary macronodular adrenal hyperplasia, **ACC** = adrenocortical carcinoma.

**Table 4:** Demographic and clinical presentation characteristics.

Parameters		Mean ± SD	Median (IQR)
**Final pathology**			
	Adenoma	59 (38.3%)	
	Pheocromocytoma	36 (23.4%)	
	PMAH	18 (11.7%)	
	ACC	11 (7.1%)	
	Other	30 (19.5%)	
**Adrenalectomy access**			
	Laparoscopic	123 (79.9%)	
	Open	25 (16.2%)	
	Converted	6 (4.9%)	
**Surgery approach**			
	Unilateral total	126	
	Bilateral total	7	
	Bilateral partial / total	21	
Surgery duration (min)		154±59	140 (120-180)
Estimated blood loss (mL)		139±224	50 (50-100)
Blood transfusion		7 (4.5%)	
Intra-operative hemodynamic instability		29 (18.8%)	
**Intra-operative complications**		**16 (10,4%)**	
	Satava 1	15 (9,7%)	
	Satava 2	1 (0,6%)	
	Satava 3	0	
**Post-operative complications**		**40 (26%)**	
	Clavien 1-2	27 (17,5%)	
	Clavien 3a	6 (3,9%)	
	Clavien 3b	3 (1,9%)	
	Clavien 4	0	
	Clavien 5	4 (2,6%)	
**Hospitalization duration (days)**		**6,1 ± 5,3**	4 (3-8)

**BMI =** body mass index; **PMAH** = primary macronodular adrenal hyperplasia; **ACC** = adrenocortical carcinoma.

Unilateral adrenalectomy was performed in 126 patients. Bilateral total adrenalectomy was done in 6 patients with Cushing's disease and 1 patient with fungal infection (agent: *Paracoccidioides brasiliensis*). Bilateral total / partial adrenalectomy was performed in 14 patients with primary macronodular adrenal hyperplasia (PMAH), 4 patients with pheocromocytoma and 3 patients with Cushing's disease. Conversion from laparoscopic to open surgery occurred in 6 cases: 3 due to hemodynamic instability aiming to abbreviate the surgery, 2 to control bleeding and 1 due to technical difficulties. Major complications occurred in 13 patients listed in Table-5. Four patients died, of whom 2 had metastatic ACC (Table-6). Results discriminated by open or laparoscopic surgery are available on Table-7.

**Table 5:** Post-operative major complications (N = 13).

Clavien classification		N
**3a (N = 6)**		
	Sepsis (respiratory)	1
	Pulmonary thromboembolism	1
	Cardiac stroke	2
	Cardiac arrhythmia	2
**3b (N = 3)**		
	Infected retroperitoneal hematoma	2
	Retroperitoneal hematoma with need of hemostasis	1
**5 (N = 4)** †		
	Septic complications	2
	Pulmonary thromboembolism	1
	Cardiac arrest	1

† Exposed with details in table 6.

**Table 6:** Cause of death (N=4).

#	Final pathology	Age (years)	Charlson score	Surgical access	Tumor size (cm)	Cause of death
1	ACC	86	6	Open	8.4	Septic shock due to pneumonie
2	ACC M+	42	8	Open	11.3	Septic shock after evisceration
3	ACC M+	55	9	Open	17.5	Cardiac arrest after severe hemodinamic instability
4	Adenoma	67	4	Lap	2.5	Pulmonary thromboembolism

**ACC** = Adrenocortical carcinoma; **M+** = Metastatic; **Lap** = Laparoscopic

**Table 7:** Results stratified by laparoscopic, open and converted surgery.

	Laparoscopic	Open	Converted
Cases	123 (79.9%)	25 (16.2%)	6 (4.9%)
Bilateral n(%)	25 (20.3%)	2 (8%)	2 (33%)
Reintervention n (%)	3 (2.4%)	2 (8%)	1 (16%)
Pathologoy			
Pheocromocytoma	32	2	2
Adenoma	56	2	1
PMAH	16	2	0
ACC	1	10	0
Other	18	9	3
Estimated blood loss (Mean ± SD)	91.2mL (± 164.8)	350mL (±374.6)	366.7mL (±206.6)
Surgery duration in minutes (Mean ± SD)	143.6 (±49.6)	208 (±80.4)	183 (±20.7)
Blood transfusion	0	6 (24%)	1 (16%)
Clavien Major (>2)	6 (4.8%)	6 (24%)	1 (16%)
Hospitalization duration in days (Median, range)	4 (2-38)	4 (1-17)	10.5 (3-17)

Univariate analysis didn't reveal association of patient sex, use of anti-coagulant drug, blood hypertension and clinical presentation with any of the outcome measures. On multivariate analysis, we found that pathological diagnosis was an independent predictor of post-operative complications (p=0.003), while pathological diagnosis (p=0.003) and patient age (p=0.004) were independent predictors of major post-operative complications. Tumor size (p<0.001) and BMI (p=0.009) were independent predictors of intra-operative complications. Pathological diagnosis (p<0.001) and Charlson score (p=0.013) were independent predictors of death. Tumor size was an independent predictor of blood-loss (p<0.001). Tumor size (p<0.001) and open adrenalectomy (p=0.005) were independent predictors of surgical time and hospitalization time, respectively. Conversion to open surgery and reoperation were not associated with any of the assessed parameters.

Historically the surgical treatment of adrenal tumors has been a worrisome topic. Four decades ago patients with Cushing syndrome had a post-operative complication risk of 30% whereas patients with pheocromocytoma had a mortality risk ranging from 30% to 45% (10). The knowledge regarding adrenal diseases evolved and consequently the associated morbidity decreased. Currently, post-operative morbidity rate in patients with Cushing syndrome is approximately 6% and mortality rate in patients with pheocromocytoma diminished to 0% to 2.9% (11). Murphy et al. reported a complication rate of 7.2% in nationwide cohort with >40.000 subjects that underwent adrenalectomy due to a variety of pathologies (2). It should be mentioned that population databases have the inherited shortcomings of coding and negligent reporting. Other series refer post-adrenalectomy complication rates ranging from 6.4% to 18.4% (4, 5). In the present cohort, despite the rate of major complications was similar to previous series (8.4%), the rate of minor complications was higher (17.5%). The elevated frequency of ACC and pheocromocytoma in our cohort might explain this finding.

## DISCUSSION

In this retrospective analysis we investigated predictors of post-operative morbidity in patients undergoing adrenalectomy due to various causes. We found that adrenal tumor etiology and size, patient age and comorbidities are predictors of post-adrenalectomy complications; open adrenalectomy prolonged hospitalization and higher BMI augmented the risk of intra-operative complications.

Minimally invasive surgery was pivotal to improve surgical outcomes. It has been demonstrated in a meta-analysis with 98 studies that complications rates after laparoscopic and open adrenalectomy were 10.9% and 25.2%, respectively (12). Bittner et al. corroborated this finding reporting a 5-fold greater risk of complications in patients undergoing open adrenalectomy (13). Of note, patients that undergo open surgery are prone to have larger tumors and to be operated in low-volume centers, what may increase complication occurrence. Excluding these cofounding factors, we found that open and laparoscopic surgery had similar complication rates. The open approach was exclusively an independent predictor for longer hospitalization. However, it is unquestionable the benefits of minimally invasive surgery regarding cosmetics, convalescence and pain. In the United Stated of America, between 1999 and 2006, 14% of adrenalectomies were performed laparoscopically (5). From 2002 to 2011 it increased to 20% (14). Adrenal surgery follows the trend toward minimally invasive procedures seen in other specialties. In this scenario, laparoscopic adrenalectomy should be preferred, with exceptions for suspected malignancy and large tumors (2, 15).

In accordance to our findings it has been shown that age and comorbidities have major influence in post-operative recovery (2, 11, 16, 17). Contemporary guidelines recommend a series of measures able to effectively minimize clinical complications, particularly in cases of Cushing syndrome, pheochromocytoma and ACC (18). Generally, patients with indication for adrenalectomy will be more prejudicated by their adrenal disease than by the potential harms of operation and thus, we cannot afford to spare most individuals from surgery. Diligent thromboembolism prophylaxis, pulmonary rehabilitation and preclusion of bowel preparation are advocated, especially in elderly, in whom the physiologic response to surgical stress is impaired (11).

Patients with ACC represent the greatest challenge in treatment management. Aside from ACC aggressiveness (median overall survival of 1.7 years), the risk of complication and mortality after adrenalectomy are 42% and 2%, respectively (19, 20). There is paucity of studies comparing the post-operative morbidity between malignant and benign adrenal tumors, however it has been shown that larger tumors are associated with higher risk of conversion and complications (13, 21, 22). Furthermore, Marcadis et al. demonstrated in a population database study that 42% of the patients with ACC had Charlson score ≥2 while that number was 17% in patients with benign adrenal lesions (23). One could expect that the clinical fragility associated with adrenal malignancy is determinant of increased morbidity. In accordance, we found that higher Charlson score and pathological diagnosis of ACC were related to greater risk of complications. Moreover, in our cohort 3 patients with malignant disease died. Despite of rigorous peri-operative control, patients with ACC still have severe post-operative complications.

The experience of the medical team is of paramount importance when treating adrenal tumors. After reviewing outcomes of 3,144 individuals that underwent adrenalectomy, Park et al. revealed that patients operated by low-volume surgeons have 1.5- fold greater risk of complications after adrenalectomy when compared to high-volume surgeons (top quartile in number of surgeries/year) (5). It has been suggested that after 30 adrenalectomies the surgeon surpass the learning curve (24). Nonetheless, it seems more plausible that frequency is more important than the absolute number of procedures performed. The proposed case-load to achieve satisfactory rates of post-operative complications varies from >3 to >30 per surgeon, yearly (25, 26). Pedziwiatr et al. reported the rate of complications of 500 adrenalectomies divided in quartiles, from first to fourth, to be 14%, 11%, 8% and 6%, respectively. Interestingly, the first and second quartiles were operated by learning surgeons, the third quartile by senior surgeons and the fourth quartile by residents. After observing the lowest rate of complications in patients operated by residents, he concluded that the growth of institutional experience was responsible for morbidity decrease (27). It has been demonstrated that when properly proctored, residents can perform adrenalectomies without raising the rates of complications (28).

This study is subject to the drawbacks of its retrospective design and relatively small sample size. We limited the research period aiming to match the last implementations in our department guidelines regarding surgical preparation for adrenalectomy, what restricted the number of patients included. However, patients were treated in a quaternary institution where >50 adrenalectomies are performed yearly and despite the participation of residents, procedures were proctored by three senior surgeons with adrenalectomy caseload >200. To our knowledge, this is the first series to compare complication rates between malignant and benign adrenal tumors and due to the complexity of the cases referred to our institution, the higher occurrence of complications allowed a convenient assessment of morbidity.

## CONCLUSIONS

Patients with adrenal tumors are at substantial risk of post-operative complications. In cases of hormone-producing or large tumors, most patients cannot be precluded from surgery. Diligent care is needed with patients harboring ACC, pheocromocytoma and large adrenal masses, who have greater risk of post-operative complications. Older age and presence of multiple comorbidities are also associated with the occurrence of post-operative complications. Open surgery was not predictor of increased morbidity, nonetheless prolonged hospital-stay. Patients with elevated BMI have higher risk of intra, but not of post-operative complications. In summary, identifying factors that influence morbidity after adrenalectomy is crucial to maximize outcomes. Our results provide knowledge to optimize surgical preparation.

## ABBREVIATIONS

ACC: Adrenocortical carcinoma
BMI: Body Mass Index
IQR: Interquartile Range
PMAH: Primary macronodular adrenal hyperplasia
